# Dexamethasone distribution characteristic following controllable continuous sub-tenon drug delivery in rabbit

**DOI:** 10.1080/10717544.2017.1324531

**Published:** 2017-05-16

**Authors:** Xuetao Huang, Manqiang Peng, Yezhen Yang, Yiqin Duan, Kuanshu Li, Shaogang Liu, Changhua Ye, Ding Lin

**Affiliations:** 1 Department of Ophthalmology, Changsha Aier Hospital, Aier School of Ophthalmology, Central South University, Changsha, China and; 2 Advanced Research Center, Central South University, Changsha, China

**Keywords:** Drug delivery, dexamethasone, topical administration, ocular tissue, rabbit eye, sub-tenon infusion, drug distribution

## Abstract

Drug delivery systems are required to be safe, minimally invasive and effectively delivery drug to the target tissues. But delivery drugs to the eye has not yet satisfied this need. Here, we focused on examining the distribution of dexamethasone (DEX) in ocular and plasmic samples following controllable continuous sub-Tenon drug delivery (CCSDD) of dexamethasone disodium phosphate (DEXP) in rabbit, and to compare that with two traditional routes: subconjunctival injection and intravenous injection. The DEX concentration was analyzed by Shimadzu LC–MS 2010 system. In CCSDD group, during observed 24 h, the mean DEX level in collected samples from highest to lowest following in order: sclera, cornea, retina/choroid, iris, plasma, aqueous humor, lens and vitreous body. In ocular solid tissue, the DEX level in posterior segment is higher than in anatomic corresponding anterior segment, but it is opposite in ocular fluid tissue. High levels of DEX were maintained at 12 h in the ocular tissue immediately after the administration. Even at 24 h, the mean DEX concentration was 31.72 ng/ml and 22.40 ng/ml in aqueous and vitreous, respectively. In CCSDD group, the ocular DEX exposure (AUC_0-24_) is much higher and plasma exposure is much less than IV group, and it is also similar in SC group except iris. The amount of DEX levels are markedly increased in ocular tissues but it yield lower plasma levels indicating reduction of systemic absorption by CCSDD. Thus, CCSDD is an effective method of delivering DEX into anterior and posterior segment of the eye.

## Introduction

An ideal drug delivery system should be safe, minimally invasive, and effective delivery of drug to the target tissues in a therapeutically effective concentration. But administering drugs to the eye has not yet met this need (Booth et al., 2007; Del & Urtti, 2008; Ali et al., 2016; Thakur et al., 2016). Because of the unique anatomic structure and the static and dynamic physiologic barriers, that prevent foreign molecules enter into the eye and protect the ocular internal tissues. As we all know that conventionally delivering drug to the anterior segment is using topical eye drops (Pokharkar et al., 2015; Abdul et al., 2016; Yellepeddi & Palakurthi, 2016), this method is generally safe and easy to operate, but drug bioavailability to the eye especially to the posterior segment including the retina is very limited (Raghava et al., 2004; Kompella et al., 2010; Yellepeddi & Palakurthi, 2016), due to the short residence time on the ocular surface, rapid elimination via precorneal tears and conjunctival blood flow, low corneal permeability together with quick flow of aqueous humor, the blood–retinal barrier and long diffusional distances from the ocular surface to the back of the eye (Maurice, 2002). Current clinical methods for drug delivery to the posterior segments are often performed via systemic delivery, which has low bioavailability and high systemic adverse effects (Zeng et al., 2016) due to drug does not target to the eye result to the limited delivery to the choroid–retina. Intravitreal injections which has excellent bioavailability, but is the most invasive with the highest risk of ocular complication rates and introduce safety concerns (Jager et al., 2004; Raghava et al., 2004; Shah et al., 2011; Kim et al., 2013). And periocular injection (Raghava et al., 2004; Ghate et al., 2007; Ranta et al., 2010; Battaglia et al., 2016; Yavuz et al., 2016), which indicates drug administration surrounding the eye including peribulbar, retrobulbar, sub-conjunctival, sub-Tenon's or posterior juxtascleral injections, and they are differ on the location of the injection in the proximity of the sclera. This is because each method has its own limitations. Taken together, transscleral avenue is an alternative choice for potential solution of the drug delivery problems, which is considered to be safer, less invasive than intravitreal injection and have been shown to be greater bioavailability than systemic drug administration for it deposits the agent adjacent to the target tissues (Geroski & Edelhauser, 2001; Raghava et al., 2004; Hsu, 2007; Thrimawithana et al., 2011; Sun et al., 2016).

Traditionally, dexamethasone is commonly used to treat inflammatory eye diseases, including superficial ocular inflammation (e.g. anterior segment uveitis, dry eye, corneal graft rejection, macular degeneration, conjunctivitis, scleritis and iritis) via topical drops, various chorioretinal diseases, noninfectious uveitis, age-related macular degeneration, diabetic macular edema, and various macular edema associated with chronic inflammation or retinal vein occlusion by system method, intravitreal and subtenon injections and so on (Coursey et al., 2015; Whitcup & Robinson, 2015; Abadia et al., 2016; Pohlmann et al., 2016). But for some acute serious ocular inflammatory disease, it need long term and large corticosteroid application to control the progress of inflammation, and led to corticosteroid mediated side effects including ocular hypertension, cataract formation, and retinal toxicity as well as systemic effects such as suppressing the hypothalamus–pituitary axis and electrolyte imbalance. Researchers have studied many strategies to overcome the aforementioned shortcomings of existing drug dosing options.

Therefore, these improve the effective quantities of drugs to the eye for treatment of ocular diseases and reduce the risk of vision threatening complications and systemic side effects. To address this need, we hypothesize a novel approach of controllable continuous sub-tenon drug delivery (CCSDD) that can deliver drugs into the eye via sub-tenon routes in a minimally invasive manner with high bioavailability and expect that this approach should minimize the risk of complications and reach to high concentrations to the ocular tissue and low in system circulation.

## Methods and materials

### Animal study

Male and female New Zealand white rabbits (supplied by the third xiangya hospital, Central South University, Changsha, China) with no ocular disease weighing 2.0–2.5 kg about 4 months old were used in this experiment. Animals were randomly allocated to three different groups, getting 6 animals per timepoint per group. The right eyes were used for experiment and the left eyes were untouched. All experiments followed the Association for Research in Vision and Ophthalmology (ARVO) Statement for the Use of Animals in Ophthalmic and Vision Research. Animals were anesthesized by intramuscularly injection of Xylazine Hydrochloride (2 ml:0.2 g, Huamu Animal Health Products Co., Jilin, 0.1–0.2 ml/kg) and topically application of Oxybuprocaine Hydrochloride Eye Drops (20 ml:80 mg, Sa, 3 times, 2 min/time) (Huang et al., 2017).

**Figure 1.**
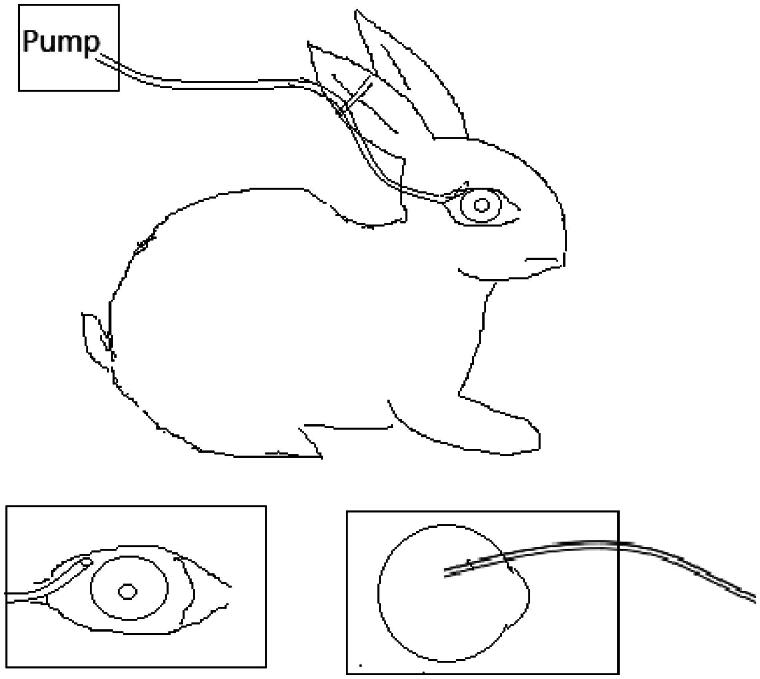
Schematic of the controllable continuous sub-tenon drug delivery (CCSDD).

There are three groups including controllable continuous sub-tenon drug delivery system (CCSDD) group, subconjunctival injection (SC) group and intravenous injection (IV) group. For CCSDD group, after anesthesia, sutured a small catheter on the scleral surface firmly, the top of the small tube is 7 mm to 10 mm posterior to the limbus (Figure 1), trickled 0.3 ml initial doses of 5 mg/ml dexamethasone disodium phosphate (DEXP, 1 ml:5 mg, Hubei Tianyao Pharmaceutical Co., Tianjin Jinyao Group, Hubei, China), connected with a pump and start timing, then constantly chronic infused DEXP into sub-tenon at a rate of 0.1 ml/h for 10 h using a pump. For SC group, we injected 0.3 ml of 5 mg/ml DEXP into sub-conjunctiva. For the IV group, we administrated 1 mg/kg DEXP intravenously into auricular vein, and then timing. At different time point within 24 h (1 h, 3 h, 6 h, 10 h, 11 h, 12 h, 16 h, 20 h and 24 h in CCSDD group. At 1 h, 2 h, 3 h, 4 h, 6 h, 8 h, 12 h and 24 h in SC group and 1 h, 3 h, 6 h, 12 h and 24 h after injection in IV group), the blood and eye samples were collected. Blood samples were obtained from auricular vein into dry heparinized tubes, and euthanized the animals by 3 ml lidocaine hydrochloride injection (5 ml:100 mg, Shanghai Chaohui Pharmaceutical Co., Shanghai, China) and 3 ml air intravenously and surgical enucleated the right eyes. Aqueous humor was collected using a 26-gauge needle, cornea, iris, lens, vitreous humor, retina/choroid, and sclera were dissected and isolated and weighted each solid tissue. The blood samples were centrifuged for 10 min at 3000*g* to extract plasma. All samples were collected in labeled polypropylene vials (Eppendorf, Hamburg, Germany), sealed, and immediately stored at −20 °C until analysis.

### Drug assay

Dexamethasone standard (100.1%, lot: 20060501) used to construct the standard curve and triamcinolone diacetate standard (99.53%) used as the internal standard (IS), were purchased from National Institute for the Control of Pharmaceutical and Biological Products (No. 2 Tiantan Xili, Beijing, China); Ultra-pure water prepared by a Millipore Milli-Q puriﬁcation system (Millipore Corp., Bedford, MA) were used as the mobile phase of HPLC–MS, and all other chemicals and solvent were of the highest analytical grade available. The concentration of dexamethasone in the samples was determined by Shimadzu LC–MS 2010 system: Pump (LC-10AD VP); Thermo Hypersil-Hypurity C18 column (150 mm × 2.1 mm, i.d., 5 μm, USA); Mobile phase (5 mM ammonium formate (pH 4.0) – methanol acetonitrile, 30:5:65, v/v/v); injection volume 5 μl. The mass selective detector (MSD) was operated in the positive ionization mode with selected-ion monitoring (SIM) at 393.6 for Dexamethasone (m/z) and 479.6 for Triamcinolone diacetate (m/z). Quantitation was performed by a linear regression analysis of peak areas ratio from a standard curve containing seven standard points (Huang et al., 2017).

For the solid samples (cornea, iris, lens, retina/choroid and sclera), mixed with 400 μl 0.9% normal saline (NS) and homogenized. For the liquid sample (plasma, aqueous and vitreous), 100 μl of the supernatant was mixed with 400 μl 0.9% normal saline (NS). Then 100 μl IS (200 ng/ml) dipped into the above mixed samples and vortex mixed for 30 s, the mixture was extracted with 1 ml n-hexane and acetate ester (1:1, v/v) for 60 min, and centrifuged at 14 000 rpm for 5 min. The organic layer was aspirated, removed and evaporated until it was dry completely. The dried residue was dissolved with 50 μlml mobile phase and centrifuged for detection.

### Pharmacokinetics and statistical analysis

The tissue concentration data are expressed in nanograms per gram or nanograms per milliliter. Pharmacokinetic parameters were analyzed with a commercial software (Kinetica 5.1; Innaphase, Philadelphia, PA) by fitting the data to the Extravascular analysis model, and non-compartmental parameters were calculated and reported. Maximum concentration (*C*_max_), time to maximum concentration (*T*_max_), elimination half-life (*T*_1/2_), the area under the concentration–time curve AUC_0–n_ were obtained. Statistical analysis was performed using SPSS release 18.0 (SPSS Chicago, IL). Dexamethasone concentrations were analyzed for normality by using K-S test and parametric or nonparametric tests were used as appropriate.

## Results

**Figure 2.**
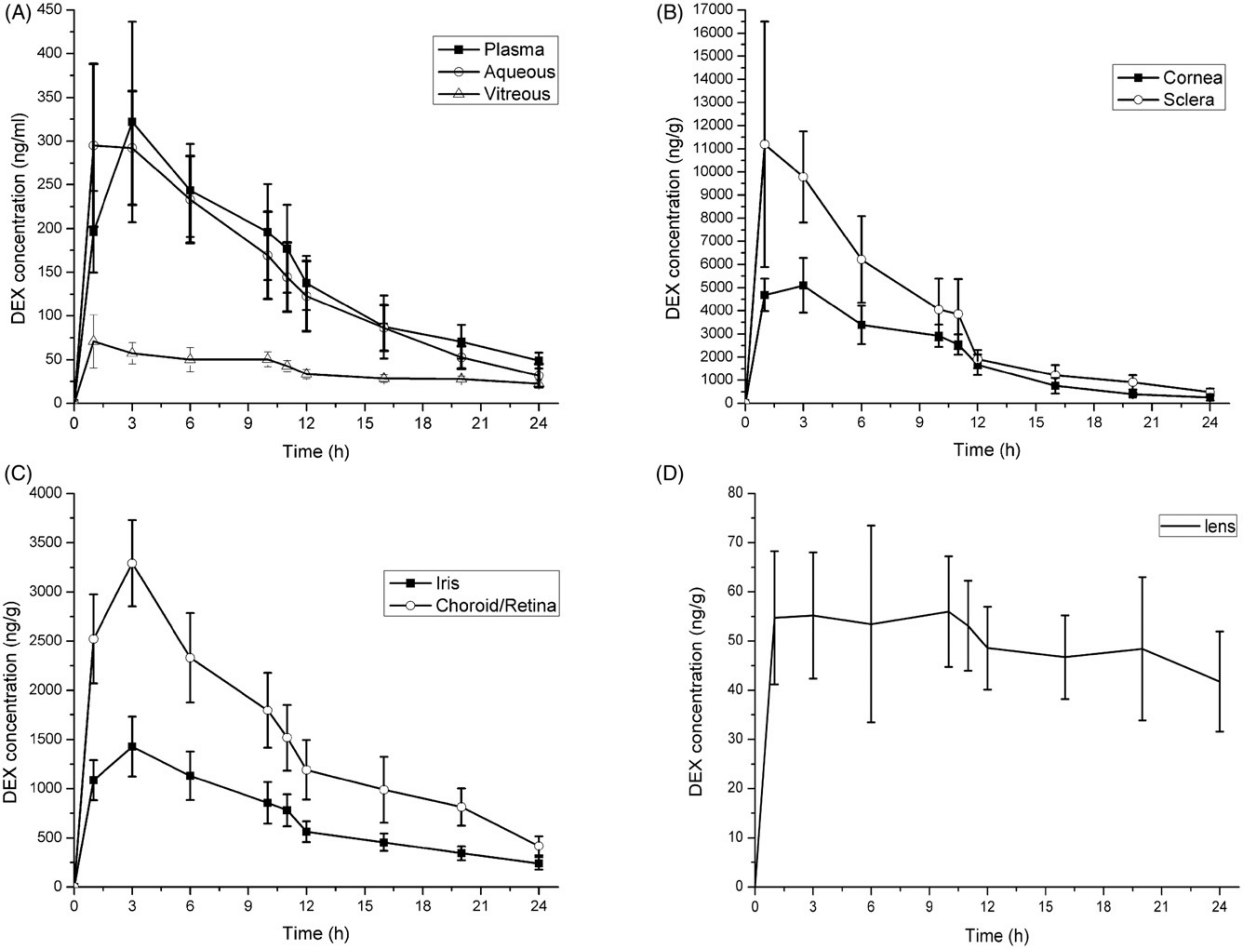
Dexamethasone concentration-time profiles in plasma and various ocular tissues in CCSDD group. (A) is in plasma, aqueous and vitreous, (B) is in corneal and sclera, (C) is in iris and choroid/retina, and (D) is in lens.

**Figure 3.**
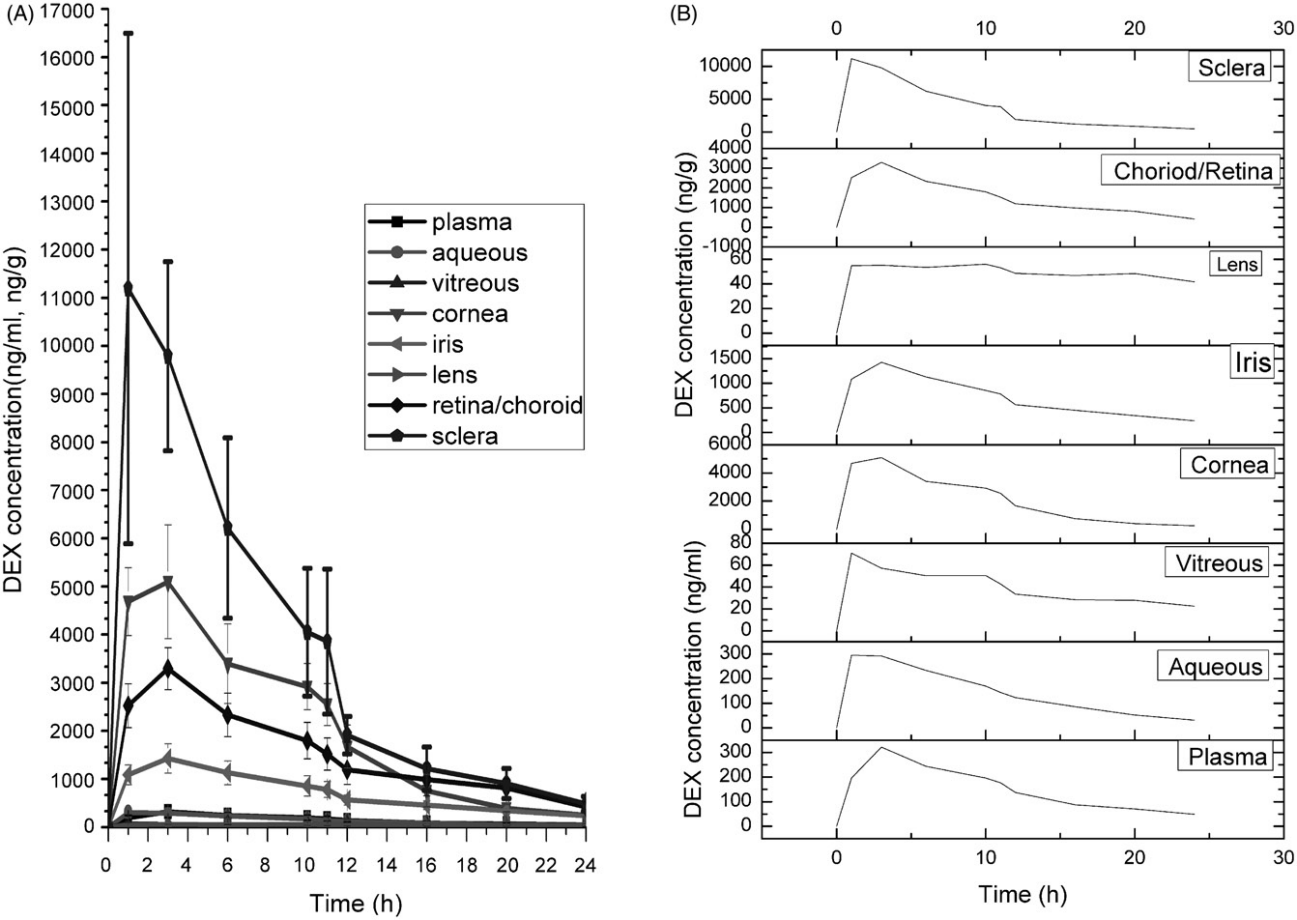
Dexamethasone concentration-time profiles in plasma and various ocular tissues in CCSDD group. (A) is in all ocular tissue and plasma, (B) is in all observed tissue in single.

In the CCSDD group, during the 24 h study, the mean DEX concentration in the samples from highest to lowest following in order: sclera (4402.58 ± 4202.89 ng/g), cornea (2413.01 ± 1793.66 ng/g), retina/choroid (1652.37 ± 935.31 ng/g), iris (764.36 ± 414.76 ng/g), plasma (164.31 ± 97.45 ng/ml), aqueous humor (158.61 ± 103.48 ng/ml), lens (50.88 ± 12.42 ng/g), vitreous body (42.67 ± 19.20 ng/ml). Scleral DEX level was higher significantly than other ocular tissues, because the drug is entry into the ocular tissue transiting through sclera, especially to the posterior segments. The DEX concentration was not significantly different in cornea and retina-choroid (*p* = 0.073), and plasma and aqueous humor (*p* = 0.610), respectively. The plasma and ocular DEX concentration–time curves in CCSDD group are shown in Figures 2 and 3, within 3 h after administration, the mean DXM concentrations have been reached the peak in plasma and ocular tissues except lens. The sclera showed the highest concentration of DEX while the cornea (within 12 h after administration) or retina-choroid (after 12 h) showed the second highest. There was also a higher amount of DEX in the iris, and a certain amount of DEX in the plasma and aqueous within 12 h. Low level of DEX was found in the lens and vitreous. High levels of DEX were observed in the ocular tissue immediately after the drug administration and was maintained at 12 h. Even at 24 h, the mean DEX concentration was 31.72 ng/ml and 22.40 ng/ml in aqueous and vitreous, respectively.

The relative relationship of DEX level in the plasma and ocular tissue of plasma/vitreous, plasma/aqueous, vitreous/aqueous, sclera/cornea and retina-choroid/iris in CCSDD group are shown in Table 1. Table 2 listed the key pharmacokinetic parameters in the three group. The maximum DEX in plasma was 321.81 ng/ml, 1798.44 ng/ml and 8441.26 ng/ml in CCSDD, SC and IV group, respectively. Each ocular tissue peak DEX level is higher in CCSDD and SC group than IV group. Although there were a similar *C*_max_ levels in ocular tissues in CCSDD and SC, the ocular exposure (AUC_0–24_) except iris to DEX is higher and plasma exposure is lower in CCSDD than SC.

**Table 1.** Relative relationship of DEX level in the plasma and ocular tissue in CCSDD group.

	1 h	3 h	6 h	10 h	11 h	12 h	16 h	20 h	24 h
Plasma/vitreous	2.76	5.62	4.85	3.88	4.13	4.11	3.07	2.54	2.19
Plasma/aqueous	0.66	1.10	1.04	1.16	1.22	1.12	1.01	1.34	1.55
Vitreous/aqueous	0.24	0.20	0.22	0.30	0.30	0.27	0.33	0.53	0.71
Sclera/cornea	2.39	1.92	1.83	1.39	1.51	1.14	1.60	2.30	1.94
Retina-choroid/iris	2.32	2.31	2.06	2.10	1.95	2.11	2.17	2.37	1.75

Relative relationship of DEX level in the plasma and ocular tissue in CCSDD group. It is the rate of liquid tissue, the rate of DEX concentration of matching posterior segment and anterior segment.

**Table 2.** Pharmacokinetic parameters of the DEX in plasma and ocular tissue.

	Plasma	Aqueous	Vitreous	Cornea	Iris	Lens	Retina/Choroid	Sclera
CCSDD group								
*C*_max_	321.81	295.18	71.05	5094.97	1426.96	55.98	3219.35	11 192.2
*T*_max_	3	1	1	3	3	10	3	1
AUC_0–24_	4201.9	3712.36	1411.65	52 012	20 044.1	3783.8	41 400.7	93 577.8
*T*_1/2_	7.52	5.54	14.32	4.99	8.62	43.12	7.65	4.89
SC group								
*C*_max_	1798.44	610.05	80.45	7410.71	2908.17	55.96	3638.8	8604.95
*T*_max_	2	2	2	2	3	2	3	1
AUC_0–24_	6091.49	2547.28	628.84	48 060.4	37 186.9	1699.77	22 411.7	35 582.3
*T*_1/2_	3.09	5.26	5.51	7.24	14.00	21.62	8.29	6.26
IV group								
*C*_max_	8441.26	28.1	43.78	303.98	341.79	8.29	284.82	402.99
*T*_max_	0	1	1	3	3	3	3	3
AUC_0–24_	13301	191.82	285.23	2252.81	2181.6	27.97	1571.71	2858.71
*T*_1/2_	2.50	2.05	4.52	4.01	3.05	–	3.85	6.88

*C*_max_(ng/ml, ng/g): maximum concentration, *T*_max_ (hour): time to maximum concentration, AUC_0–24_ (ng*h/g, ng*h/ml): area under the concentration–time curve between 0 and 24 hour, *T*_1/2_ (h): elimination half-life.

The relative relationship of DEX levels in all corresponding samples among CCSDD group, SC group and IV group were summarized in Table 3. Plasmic drug levels were lower within 6 h and ocular samples (except iris) DEX levels were equal or higher in CCSDD group than that in the SC group. Meanwhile plasmic drug levels were lower within 12 h and all ocular samples DEX levels were higher within observed time in CCSDD group than that in the IV group.

**Table 3.** Relationship of DEX levels in all corresponding samples of CCSDD/SC and CCSDD/IV.

	1 h	3 h	6 h	12 h	24 h
CCSDD group/SC group					
Plasma	0.23	0.36	0.70	1.55	–
Sclera	1.30	2.21	3.78	4.13	2.04
Cornea	0.88	1.12	1.33	1.71	0.52
Retina/Choroid	1.34	0.90	1.75	2.89	2.27
Iris	0.81	0.49	0.73	0.62	0.44
Aqueous	0.79	0.81	1.91	2.58	2.82
Vitreous	1.12	0.78	1.45	1.48	6.04
Lens	1.49	1.04	1.24	1.64	1.49
CCSDD group/IV group					
Plasma	0.10	0.23	0.41	0.66	15.17
Sclera	39.77	24.28	43.90	29.73	21.80
Cornea	27.11	16.76	21.72	24.01	32.94
Retina/Choroid	17.94	11.56	25.74	30.60	112.72
Iris	5.46	4.17	7.46	9.09	89.25
Aqueous	10.50	11.14	10.39	82.91	–
Vitreous	1.62	1.90	2.33	6.28	16.35
Lens	11.45	6.66	31.26	–	–

The description of the different ocular tissue and plasma dexamethasone concentration level ratio among CCSDD and SC group, CCSDD and IV group.

–delete the denominator is zero.

## Discussion

Glucocorticosteroids, such as dexamethasone, are widely used in various ocular inflammatory disorders (Hunter & Lobo, 2011; Abadia et al., 2016). Their use typically requires achieving significant drug levels in the anterior and/or posterior segments of the eye (Abadia et al., 2016). Sub-tenon way is gaining a high level of concern because of its safety, easy penetration, large area and minimal invasion (Nan et al., 2010; Bondalapati & Cabrera, 2014). In this study, we designed a controllable continuous sub-tenon drug delivery system, which can deliver DEXP at a chronic controllable constant rate. CCSDD processing can be repeated every day, which was similar as intravenous injection. Our study found that the way of CCSDD can reach a higher DEX concentration in ocular tissue and lower DEX level in plasma comparing with SC and IV, and can keep a relatively higher level in ocular tissue within observed 24 h.

In CCSDD group, DEX levels of sclera and retina-choroid in posterior segment are higher than matching cornea and iris in anterior segment, while vitreous DEX levels are lower than corresponding aqueous. The sclera showed the highest concentration of DEX while the cornea (within 12 h after administration) or retina-choroid (after 12 h) showed the second highest. It demonstrate that the clearance of DEX is quicker in cornea than retina-choroid, it may be owing to the DEX connecting with choroid or retinal and the elimination is slower. The sclera DEX level was higher than cornea or retina-choroid because the drug is connected with it and penetrates it directly. We speculated that the DEX came from the blood vessels of corneal limbs to cornea or the DEX permeated to the sclera then to the root of the cornea or penetrate to choroid. Higher retina-choroid DEX level was found than iris owing to the choroid is adjacent to the sclera, which has good drug permeability and the drug came to choroid directly. The drug reach to the iris from posterior segment of choroid or the sclera to the base of the iris. But the aqueous DEX level was higher than vitreous, it may be explained by the retinal pigment epithelium barrier and internal limiting membrane preventing drugs to vitreous, and the drug leakage from the sub-tenon intubation point and translate through corneal into anterior chamber. The *T*_max_ in sclera, aqueous and vitreous was shorter than in other ocular tissues in CCSDD group, which demonstrate that DEX entered into them more rapidly, and the velocity was slower and similar in other tissue including plasma, cornea, iris and retina/choroid. In all observed tissue, there was an obvious absorption phase at the first hour. Within the 24 h observed time of CCSDD group, the DEX concentration was the highest in sclera, then in retina/choroid and cornea which were similar, followed by in iris, plasma, aqueous, lens and vitreous in order; meanwhile DEX concentrations in aqueous and plasma were similar. We speculated that the drug is located directly on the scleral surface, then came from sclera to cornea or choroid, then to the root of the iris. There was a relatively higher concentration of DEX at the cornea, indicating that may be some amount of DEX got into the aqueous from the cornea.

In the posterior segment of the CCSDD group, the DEX concentration in the retina/choroids was just second to the sclera among the ocular tissue in this experiment. The drugs have to permeate several layers such as sclera, choroids-Bruch’s membrane and retinal pigment epithelium to reach the neuroretina (Pescina et al., 2012). The transscleral intraocular tissue distribution of corticosteroids was primarily driven by the drug solubility (Thakur et al., 2011). And DEXP is a water soluble phosphate of dexamethasone can easily get through the sclera into the choroids. Meanwhile, we just find low concentration of DEX in the vitreous, may be owning to the retinal pigment epithelium barrier and integrated inner limiting membrane, a basement membrane, which acts as a border between the vitreous humor and the retinal neuroretina (Dalkara et al., 2009; Boye et al., 2016). Overall, the high concentrations of the DEX in the choroids and retina ensure that the CCSDD system can act as an alternative choice to the posterior drug delivery. The free DEXP released from the CCSDD on the episclera might penetrate the sclera and diffuse into the choroid/retina and vitreous by concentration gradient force. This was suggested by the decrease of DEX concentration from sclera to the vitreous. But it seems opposite to the work by Zhen Huang et al. (2016), which observed a much higher DEXP concentration in the choroid and retina, then in the sclera, and the lowest concentration in the vitreous after the DEXP post sub-tenon implant. The reason has not yet been made clear. It may be associated with the release rate of DEXP in two different types of drug delivery system, different sites of sampling and the total amount of choroids and retina is much lower than the sclera. In the anterior segment of the rabbit’s eye, high concentration of DEX was found at the iris, just second to the cornea. Although being part of the anterior segment, there were relatively low concentrations of DEX in aqueous humor indicating that may be small amount of DEX got into the anterior chamber or the fast turnover of aqueous humor, which wash away the drug. In the CCSDD group, DEX distributed to all ocular tissues and were measurable till 24 h at a higher concentration. And in solid tissue, DEX concentrations were higher compared to liquid tissue except lens. All the ocular tissue concentrations are expected to be within the therapeutic window. DEX could be present in circulation after administration in all group, systemic exposure of DEX were lower in CCSDD group than two control group, which may be associated with the constant gradual delivery that permits more complete scleral absorption because the drug sclera contact time is greater than the lag time to steady state flux. AUC_0–24_ was 4201.9 ng*h/ml, 6091.49 ng*h/ml and 13301 ng*h/ml in CCSDD, SC and IV group, respectively, in plasma. Even though some amount of DEX was found in the serum, it was lower than in SC and IV group; it means that rarely systemic side effect will be occurred by administration of CCSDD. Though systemic drug concentrations are minimum in CCSDD group, it still can be measured, and thus systemic side effects should be considered when used repeatedly.

Even though subconjunctival injection is the most popular and easiest method applied by the ophthalmologists, it does not keep higher drug level for a longer time. And intravenous administration is a major method for posterior segment disease, but it has systemic side effect and do not reach targeted tissue. So, we hypothesize that CCSDD system can keep continuous administration and higher drug level in ocular tissue. Compared to subconjunctival injection, the CCSDD has priority in the relatively sustained higher DEX concentrations at least for 20 h, meanwhile it is only lasted for 6 h in SC injection. This was consistent with the former report that DEX can reach the peak at two or three hours after injection of DEXP in different ocular tissues (Weijtens et al., 1999). The DEX concentration is very low in the IV group especially at the anterior segment, the peak concentration time is 1 h in aqueous and vitreous and 3 h in other ocular solid tissue. In this study, we can see that it can reach a high DEX concentrations in the ocular tissue and low in the plasma in CCSDD group comparing with the SC and IV group. In the iris, the DEX concentration is lower in CCSDD group than SC group, it may be caused by the site of the administration, which is near to the limbus in the SC group and the drug leakage from the sub-tenon implant point.

Species (human and rabbit) and disease state may impact on the DEX level. First, there were differences in the anatomy between the human and rabbit eye, the rabbit eye is smaller and sclera is thinner than the human eye, which facilitates DEX movement into the choroid. Meanwhile rabbits have a higher blood circulation rate and choroidal flow rate, which may lead to a shorter half-life of DEX in various ocular tissues and systemic circulation. Rabbits have a much smaller body weight than humans, so the same amount of DEX administered in humans might result in much lower or undetectable concentration of DEX in the circulation. Though the relative relationship of DEX levels among the intraocular tissues should be similar, it is difficult to extrapolate PK data obtained from animal experiments to humans. Furthermore, the DEX levels in each ocular tissues cannot be measured *in vivo* at different time point at the same one owing to the solid ocular specimen only be taken at a single time point in each animal. Lastly, it is only performed in normal animals and our research group do further conformed research in experimental diseased animal models such as experimental autoimmune uveitis. Moreover, we only did the preliminary study of the CCSDD. It needs the specialized needling instrument and sub-miniature pump, which will be using in our future work.

## Conclusion

The current study exhibits the DEX pharmacokinetics in ocular tissues and plasma after a single CCSDD administration. And it can yield a high DEX level in local ocular tissues at the same time low level in the systemic circulation. So it can act as an alternative choice for the transscleral drug delivery to anterior and posterior segment of the eye.
